# Exploring the mechanism of action of Modified Simiao Powder in the treatment of osteoarthritis: an *in-silico* study

**DOI:** 10.3389/fmed.2024.1422306

**Published:** 2024-10-18

**Authors:** Zhouhengte Xu, Pingping Su, Xiahui Zhou, Zhihui Zheng, Yibo Zhu, Qinglai Wang

**Affiliations:** ^1^Wenzhou TCM Hospital of Zhejiang Chinese Medical University, Wenzhou, China; ^2^The Third School of Clinical Medicine, Zhejiang Chinese Medical University, Hangzhou, China

**Keywords:** traditional Chinese medicine, network pharmacology, pathway, molecular docking, molecular dynamics simulation, integrative medicine, osteoarthritis

## Abstract

**Introduction:**

Osteoarthritis (OA) is the most common form of arthritis and the leading musculoskeletal disorders in adults. Modified Simiao Powder (MSMP) has been widely used in the treatment of OA with remarkable clinical ecaciousness.

**Objective:**

This study aimed to elucidate underlying mechanisms of MSMP in OA by employing network pharmacology, molecular docking, and molecular dynamics simulations, due to the unclear mode of action.

**Methods:**

Bioinformatic analysis was used to evaluate the major chemical constituents of MSMP, determine prospective target genes, and screen genes associated with OA. Network pharmacology methods were then applied to identify the crucial target genes of MSMP in OA treatment. Further analyses included gene ontology (GO) and Kyoto Encyclopedia of Genes and Genomes (KEGG) pathway enrichment. These key targets within the pertinent pathways was further confirmed by molecular docking, binding energy evaluation, and molecular dynamics simulations.

**Results:**

Network pharmacology analysis identified an MSMP component–target–pathway network comprising 11 central active compounds, 25 gene targets, and 12 biological pathways.

**Discussion:**

These findings imply that the therapeutic effects of MSMP was potentially mediated by targeting several pivotal genes, such as androgen receptor (*AR*), *NFKB1, AKT1, MAPK1*, and *CASP3*, and regulating some pathways, including lipid metabolism and atherosclerosis, the AGE–RAGE signaling pathway in diabetic complications, the PI3K–Akt signaling pathway, fluid shear stress, atherosclerosis, and Kaposi's sarcoma-associated herpesvirus infection. Molecular docking assessments demonstrated that these compounds of MSMP, such as berberine, kaempferol, quercetin, and luteolin, exhibit high binding anities to AR and AKT1. Molecular dynamics simulations validated the interactions between these compounds and targets.

**Conclusion:**

The therapeutic effect of MSMP likely attributed to the modulation of multiple pathways, including lipid metabolism, atherosclerosis, the AGE-RAGE signaling pathway, and the PI3K-Akt signaling pathway, by the active components such as berberine, kaempferol, luteolin, and quercetin. Especially, their actions on target genes like AR and AKT1 contribute to the therapeutic benefits of MSMP observed in the treatment of OA.

## 1 Introduction

Osteoarthritis (OA) is a degenerative joint condition characterized by progressive joint cartilage erosion, aberrant bone proliferation, synovitis, and joint pain, which affects the lives of many individuals in later years (1). The pathological manifestations of OA include cartilage deterioration, subchondral bone sclerosis, and synovitis (2). OA has been the most common form of arthritis and the leading musculoskeletal disorders in the word, which affects approximately 22% of adults and with an incidence of 49% among those over 65 (3).

The exploration of OA within the framework of Traditional Chinese Medicine (TCM) has a millennial history (4). TCM has made substantial contributions to global health through its holistic approaches and individualized treatment strategies (5). TCM theory addresses OA by “nourishing qi and blood, enhancing blood flow to eliminate stasis, and fortifying the liver and kidney functions.” With the accumulation of extensive theoretical knowledge and therapeutic practices over the centuries, TCM possesses substantial potential for the treatment of OA (6). Originating from the Qing dynasty's “Cheng Fang Bian Du,” Simiao Powder is a TCM that comprises *Atractylodes lancea* (Atractylodis Macrocephalae Rhizoma)*, Phellodendron amurense* (Phellodendri Amurensis Cortex)*, Achyranthes bidentata* (Achyranthis Bidentatae Radix), and *Coix lacryma-jobi* (Coicis Semen). This TCM formula, dated back to “Danxi Xinfa” (1347), has been used to alleviate OA symptoms for centuries. Over decades of clinical practice, Professor Qinglai Wang from the Zhejiang Chinese Medical University has made notable advancements in the treatment of OA through the use of Modified Simiao Powder (MSMP). The modified formula of MSMP is composed of nine herbs: *Atractylodes lancea* (Atractylodis Macrocephalae Rhizoma)*, Phellodendron amurense* (Phellodendri Amurensis Cortex)*, Achyranthes bidentata* (Achyranthis Bidentatae Radix)*, Coix lacryma-jobi* (Coicis Semen)*, Lonicera japonica* (Japanese honeysuckle vine)*, Retinervus luffae fructus* (Luffa vegetable sponge)*, Ligusticum chuanxiong* (Chuanxiong Rhizoma)*, Lycopodium japonicum* (Common japanese clubmoss), and *Liquidambar formosana* (Beautiful sweetgum). Though these nine TCM components or their compounds are promising candidates for OA treatment, the understanding of the specific mechanisms and targets of Modified Simiao Powder (MSMP) in managing OA remains limited and lacks comprehensive insights. Thus, employing creative strategies and fresh perspectives is vital for a comprehensive understanding of the anti-OA mechanisms of MSMP (7).

Owing to the lack of modern research methodologies, TCM has often been perceived as empirical medicine (8). Network pharmacology (NP) has introduced a novel methodological paradigm for comprehensively understanding traditional medicines, leading to the emergence of Traditional Chinese Medicine Network Pharmacology (TCM-NP) (9). As an interdisciplinary amalgamation of traditional and contemporary medicine, information science (10), and systems science, TCM-NP has been advancing since 1999 with the formulation of initial hypotheses (11) and further refinement in 2002 (12), predating the term “network pharmacology” introduced in 2007 (13). The principles of TCM-NP align well with the holistic approach and individualized treatment of TCM, positioning it at the forefront of traditional medicine research (14). Molecular docking, a sophisticated computational method, is extensively employed in drug design and discovery to predict the binding modes and energies between receptors and ligands, thus identifying optimal drug–ligand interactions (15). This technique elucidates receptor–ligand molecular interactions and aids in the pharmacological evaluation of TCM formulations and individual herbs (16). Moreover, molecular dynamics (MD) simulations provide a detailed temporal analysis of biological macromolecules at the atomic level, which may have great value in validating highquality computational studies in bioinformatics (62). For example, MD simulations have revealed disulfiram-induced zinc ejection mechanisms for the nonstructural protein 5A of the Hepatitis C virus (60), which elucidates the therapeutic potential of disulfiram (17). Nasir et al. (18) conducted MD simulations to investigate the interactions between the receptor-binding domain of the SARS-CoV-2 spike protein (wild-type and omicron variant) and key proteins located at the blood–brain barrier. Molecular dynamics simulations and molecular docking are closely related and are often used in conjunction to enhance the reliability of results (19). The structural and dynamic insights offered by MD simulations provide valuable information for guiding future drug designs. Consequently, the use of Network pharmacology to identify and predict active MSMP components is highly promising.

**Figure 1:**
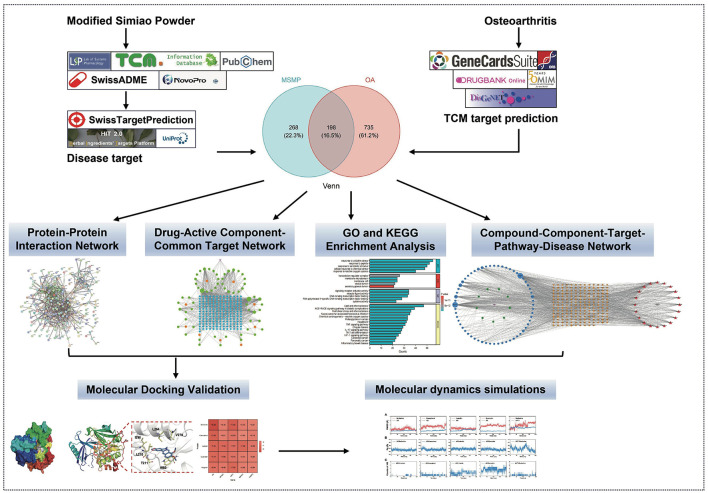
Methods overview of elucidating the potential of Modified Simiao Powder in combating osteoarthritis.

Construction of a “drug–component–target–pathway–disease” interactive network, coupled with gene ontology (GO) and Kyoto Encyclopedia of Gene and Genome (KEGG) pathway enrichment analyses, molecular docking, and MD simulations, explored the potential molecular mechanisms of MSMP in OA treatment (Figure 1). This study offers valuable insights for future fundamental experimental studies.

## 2 Materials and methods

### 2.1 MSMP active component and target selection

The medicinal herbs in the MSMP prescription were entered into the Traditional Chinese Medicine Systems Pharmacology Database and Analysis Platform (TCMSP: https://old.tcmsp-e.com/tcmsp.php) to select active components with at least 30% oral bioavailability (OB) and 0.18 drug-likeness (DL); (20). For herbs absent from the TCMSP, the Traditional Chinese Medicine Information Database (TCM-ID: https://bidd.group/TCMID/index.html) was used. Compounds not represented in TCMSP underwent further evaluation using the SwissADME database (http://www.swissadme.ch/), based on a “high” gastrointestinal absorption (GI absorption) score and minimum two “Yes” indications for drug-likeness (21). The canonical SMILES data for all selected active components were initially sourced from PubChem (https://pubchem.ncbi.nlm.nih.gov/); for those unlisted, the NovoPro tool (https://www.novopro.cn/tools/mol2smiles.html) served to generate this information.

The HIT database (http://hit2.badd-cao.net) and SwissTargetPrediction (http://www.swisstargetprediction.ch/) were used to identify corresponding gene targets. Final target validation and corrections were performed using the UniProt database (https://www.uniprot.org/), securing gene names and UniProt IDs while excluding non-human genes to identify the potential targets of the MSMP active components.

### 2.2 Potential disease target identification

Employing “osteoarthritis” as the keyword, disease-related target information was collected from databases such as Gene Cards (https://www.genecards.org/), Drug Bank (https://go.drugbank.com/), DisGeNET (https://www.disgenet.org/), Online Mendelian Inheritance in Man (OMIM: https://www.omim.org/), and the Therapeutic Target Database (TTD: https://db.idrblab.net/ttd/). The compiled targets from these databases were merged and duplicates were eliminated and validated using the UniProt database to construct a comprehensive disease target profile.

### 2.3 Protein–protein interaction network development

R software (version 4.2.1) facilitated the selection of targets shared by the MSMP and OA, which were then uploaded to the STRING database (https://cn.string-db.org/) for protein–protein interaction (PPI) network construction. The specified biological species was “*Homo sapiens*,” with the confidence level threshold set to “highest confidence” (≥0.900), and default settings maintained for other parameters. The obtained TSV file was subsequently used for network visualization using Cytoscape (version 3.10.1; https://cytoscape.org/).

### 2.4 Drug–active component–common target network construction

Using Cytoscape software (version 3.10.1), a network illustrating the interrelations between the active compounds and their targets was established. The “CentiScaPe 2.2” plugin within this software analyzed the network topological features, calculating parameters such as “Degree,” “Closeness,” and “Betweenness” to highlight pivotal compounds.

### 2.5 GO and KEGG enrichment analysis

The org.Hs.eg.db package served as the annotation library in R, with the clusterProfiler package (version 4.4.4) for ID conversion and enrichment analyses of shared target genes (22). The ggplot2 package (version 3.3.6) was used to represent the results graphically.

### 2.6 Compound–component–target–pathway–disease network development

Cytoscape software (version 3.10.1) was used to create a network that included compounds, components, targets, pathways, and disease associations. The “CentiScaPe 2.2” plugin was re-applied to assess the topological characteristics and compute “Degree” values. Nodes with elevated “Degree” values were deemed central to the potential therapeutic impact of MSMP on OA.

### 2.7 Molecular docking validation

The complete names of the principal compounds were retrieved from the UniProt IDs in the UniProt database and the corresponding protein 3D structures (AR: 2PNU, NFKB1:1SVC, AKT1:4EJN, MAPK1:3W55, and CASP3:3H0E) were downloaded from the RCSB PDB database (https://www.rcsb.org/); (23). Water molecules were removed from the receptor proteins using the PyMOL software (version 2.5.8); (24). Subsequently, the 3D molecular structures of the key target genes were acquired from the PubChem database, and the OpenBabel software (version 2.3.2) was used to convert the SDF files to the mol2 format (25). The proteins were subsequently prepared by adding hydrogen atoms and calculating charges using AutoDockTools (version 1.5.7); the target genes were annotated, and molecular docking was conducted (26). Finally, binding-energy heat maps were created using the ggplot2 package in R software (version 4.2.1), and the top five docking results were visualized using PyMOL software (version 2.5.8).

### 2.8 MD simulations

The highest-ranked AutoDock model served as the starting point for the MD simulations. Amber ff14sb (27) and GAFF (28) force fields were used to characterize the proteins and ligands, respectively. For each ligand, a single-point task was executed using the ORCA software (version 5.0.4) with the B3LYP(D3)/def2-TZVP basis set, and RESP2 charges (29) were computed using the Multiwfn software (30). The Sobtop tool (v.3.1) was used to generate topology files of the ligands.

MD simulations were conducted using GROMACS software (version 2023.2) (31). Each complex was solvated using TIP3P water models, and 0.15 M sodium and chloride ions were added to neutralize the system. Following energy minimization, the temperature of the system was gradually increased from 50 to 300 K over 200 ns, succeeded by 1-ns of NPT equilibration at a pressure of 1 bar. Subsequently, 200-ns production MD simulations were performed for each system at 300 K and 1 bar. A time step of 2 fs was used for all simulations. Cutoff distances for van der Waals and short-range electrostatic interactions were 12 Å. The LINCS algorithm (32) constrained hydrogen bonds, whereas the temperature and pressure were maintained using the V-rescale (33) and C-rescale (34) methods, respectively.

## 3 Results

### 3.1 Identification of active components in MSMP and their corresponding targets

After consolidating the data from the TCMSP and TCM-ID databases and removing duplicates, we identified 119 potentially active compounds derived from various sources: *Atractylodes lancea* (9 compounds), *Phellodendron amurense* (37 compounds), *Achyranthes bidentata* (20 compounds), *Coix lacryma-jobi* (9 compounds), *Lonicera japonica* (25 compounds), *Retinervus luffae fructus* (3 compounds), *Ligusticum chuanxiong* (7 compounds), *Lycopodium japonicum* (5 compounds), and *Liquidambar formosana* (4 compounds). Notably, wogonin, quercetin, berberine, sitosterol, beta-sitosterol, and stigmasterol were frequently observed. Using the HIT and SwissTargetPrediction databases for predictive analyses, and subsequent validation and refinement using the UniProt database, we ultimately identified 466 potential targets from *Atractylodes lancea* (62 targets), *Phellodendron amurense* (353 targets), *Achyranthes bidentata* (388 targets), *Coix lacryma-jobi* (77 targets), *Lonicera japonica* (321 targets), *Retinervus luffae fructus* (5 targets), *Ligusticum chuanxiong* (10 targets), *Lycopodium japonicum* (28 targets), and *Liquidambar formosana* (19 targets). For further information, please refer to the Supplementary material.

### 3.2 Potential OA-associated target genes

We gathered OA-related target information from Gene Cards, Drug Bank, DisGeNET, OMIM, and TTD databases, ranked by score values. After two rounds of median filtering and excluding targets below this threshold, we identified 1,665, 192, 924, 30, and 32 target genes from each database. Synthesis and adjustment using the UniProt database yielded 933 OA target genes. For further information, please refer to the Supplementary material.

### 3.3 PPI network construction

**Figure 2:**
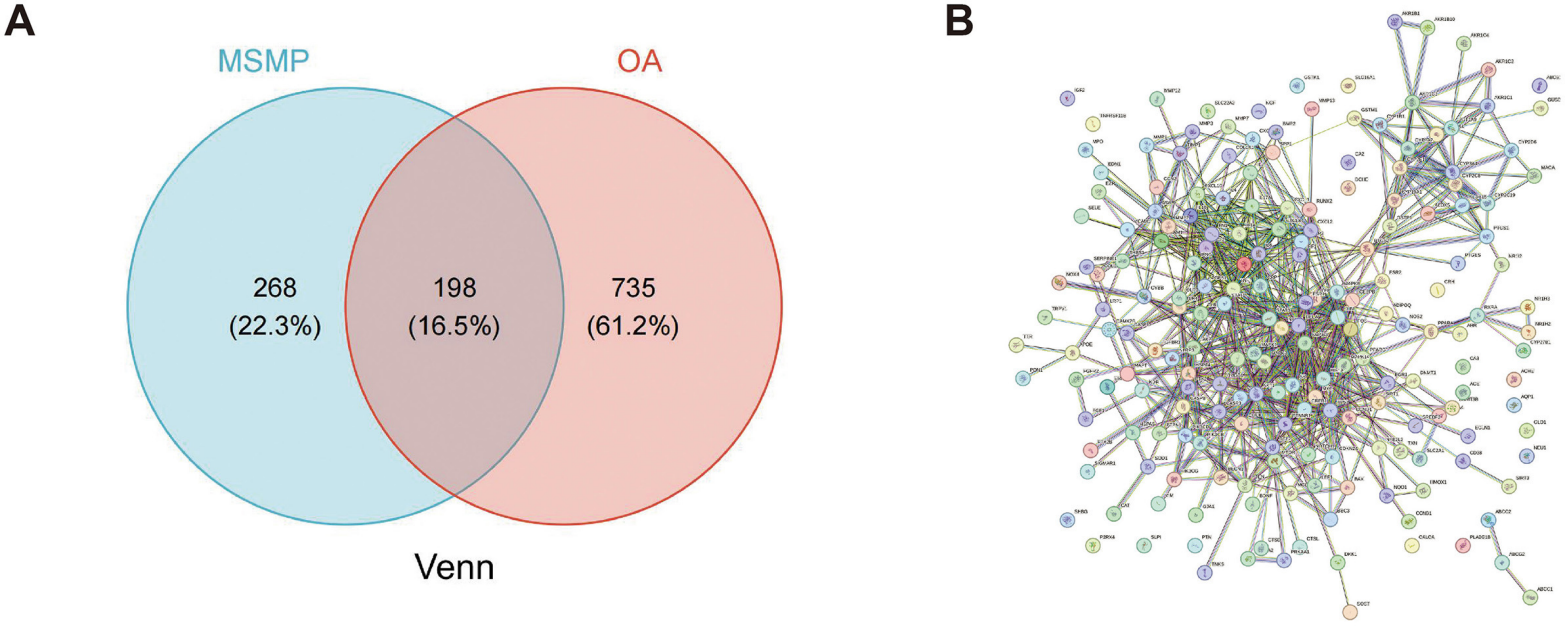
Intersection targets of Modified Simiao Powder and osteoarthritis. **(A)** Venn diagram; **(B)** STRING database protein–protein interaction (PPI) network (confidence score ≥ 0.900).

**Figure 3:**
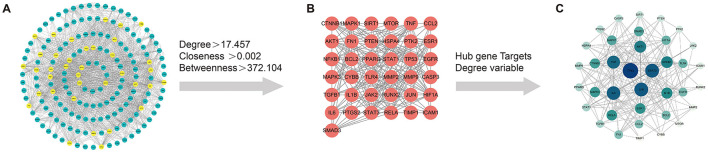
Screening process of intersection core targets of Modified Simiao Powder and osteoarthritis. **(A)** Intersection target network diagram, yellow represents the core targets screened through DC, CC, and BC; **(B)** Diagram of the network of 37 core targets; **(C)** Sorted by Degree value size, Color intensity and circle size represent the Degree value.

The initial Venn diagram analysis revealed 198 targets shared between MSMP and OA, indicating potential therapeutic targets of MSMP in OA (Figure 2A). These targets were input into the STRING database with the Required score set to “highest confidence 0.900,” and the results were saved in PNG format (Figure 2B). The TSV format data were analyzed using the Cytoscape software (v3.10.1), revealing 176 genes and 1,514 edges. After excluding three marginally relevant genes, the final network comprised 173 genes and 1,510 edges (Figure 3A). The “CentiScaPe 2.2” plugin identifies key active components based on the median threshold of degree values, closeness, and betweenness centrality, yielding 37 central targets and 390 edges (Figures 3B, C). For further information, please refer to the Supplementary material.

### 3.4 Drug–active component–common target network establishment

**Figure 4:**
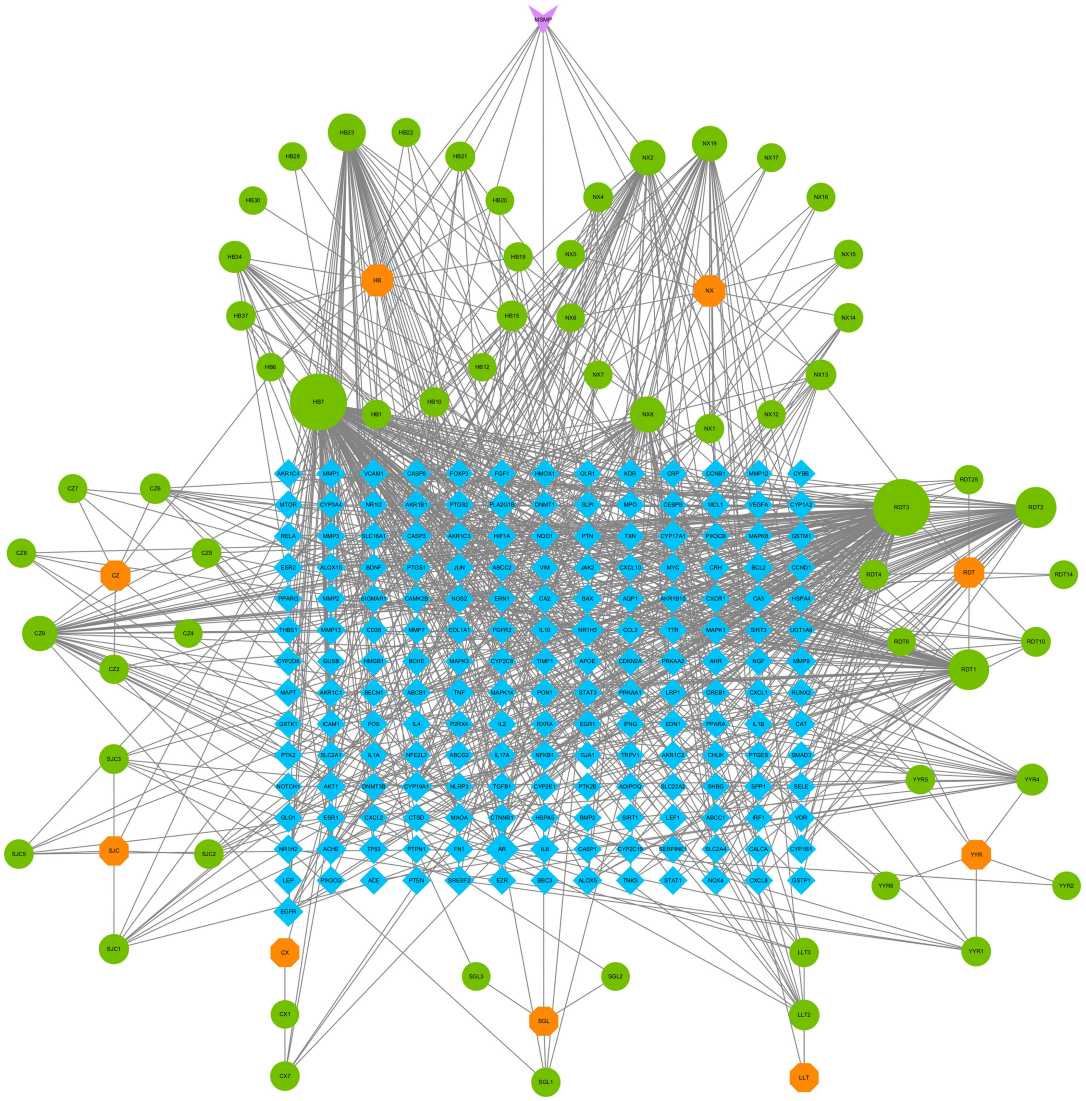
Chinese herbal compound–drug–active component–common target network. Among them, the purple inverted triangle represents the Modified Simiao Powder (MSMP), the orange hexagon represents the Traditional Chinese medicine, the green circle represents the active ingredient, and the blue rectangle represents the target. The figure size indicates the Degree value.

To elucidate the interplay between the active components of MSMP and their corresponding target genes, we constructed a drug–active component–target network (Figure 4). This network consisted of 253 nodes (1 compound prescription name, 9 Chinese herbal medicine names, 60 active components, and 183 targets) and 842 edges. The key active components were filtered using the “CentiScaPe 2.2” plugin, based on a benchmark of twice the median degree value and proximity to median CC and BC values. Active compounds such as quercetin, luteolin, kaempferol, berberine, and wogonin demonstrated high degree values, highlighting their potential significance in OA treatment using MSMP (Table 1).

**Table 1:** Screening of core compounds.

**Chemical component**	**Degree**	**Closeness**	**Betweenness**
Quercetin	133	0.002252252	18,544.38593
Luteolin	59	0.001628664	7,345.076723
Kaempferol	58	0.001623377	3,457.295087
Berberine	45	0.001602564	7,957.598243
Wogonin	33	0.001453488	1,534.604722

### 3.5 GO and KEGG pathway enrichment analysis

**Figure 5:**
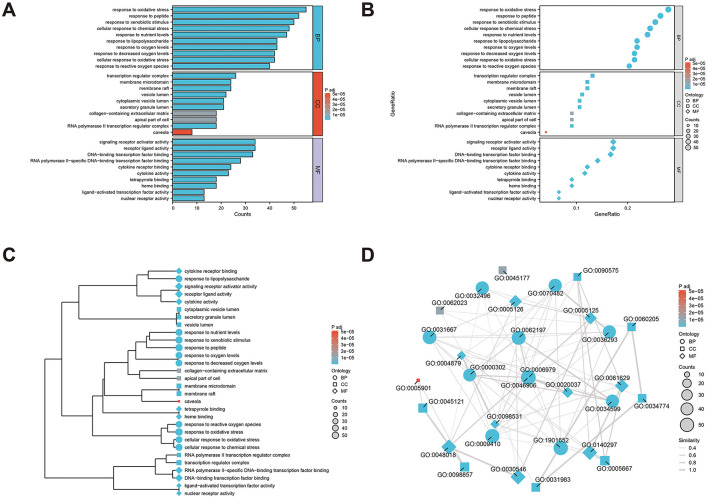
Gene ontology (GO) analysis of the core anti-osteoarthritis targets of Modified Simiao Powder (MSMP; *P* < 0.05), and top 10 biological processes (BP), cellular components (CC) and molecular functions (MF) were selected. **(A)** bar graph; **(B)** bubble diagram; **(C)** dendrogram; **(D)** EMAP diagram.

**Figure 6:**
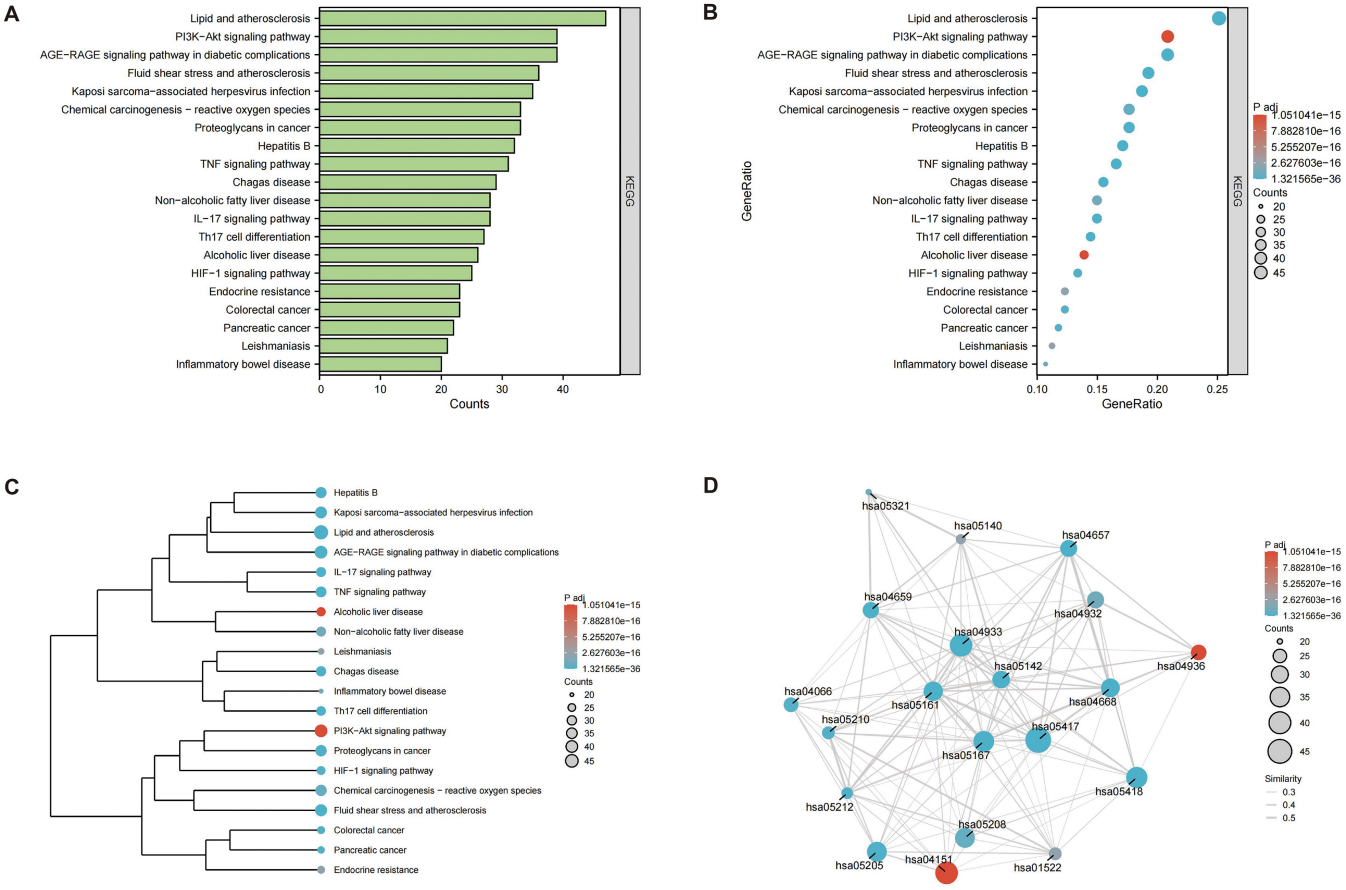
Kyoto Encyclopedia of Gene and Genome (KEGG) enrichment pathway analysis (*P* < 0.05), the top 20 highly enriched pathways were selected. **(A)** bar graph; **(B)** bubble diagram; **(C)** dendrogram; **(D)** EMAP diagram.

To clearly understand the roles of the intersecting genes, R software (v4.2.1) was used for GO functional enrichment and KEGG pathway enrichment analyses of the 198 intersecting targets, adopting a significance threshold of *P* < 0.05. GO analysis revealed that biological processes (BP) predominantly encompassed antioxidant activities, cellular responses to chemical stimuli, and reactions to reactive oxygen species; cellular components (CC) were primarily membrane rafts, microdomains, and vesicles; and molecular functions (MF) mainly involved receptor ligand and signal receptor activator activities, as well as DNA-binding transcription factor interactions. The top 10 results for BP, CC, and MF were visualized using the ggplot2 package (3.3.6) in various chart forms: bars, bubbles, dendrograms, and EMAP (Figures 5A–D). KEGG analysis identified 182 enriched pathways (*P* < 0.05), with prominent pathways including AGE–RAGE signaling, lipid and atherosclerosis, Chagas disease, tumor necrosis factor (TNF), IL-17, and HIF-1 signaling pathways. The top 20 pathways were visualized using the ggplot2 package (Figures 6A–D). These findings suggest that TCM exerts its therapeutic effects on OA initiation, progression, and prognosis through a multitude of targets and pathways.

### 3.6 Compound–component–target–pathway–disease network construction

**Figure 7:**
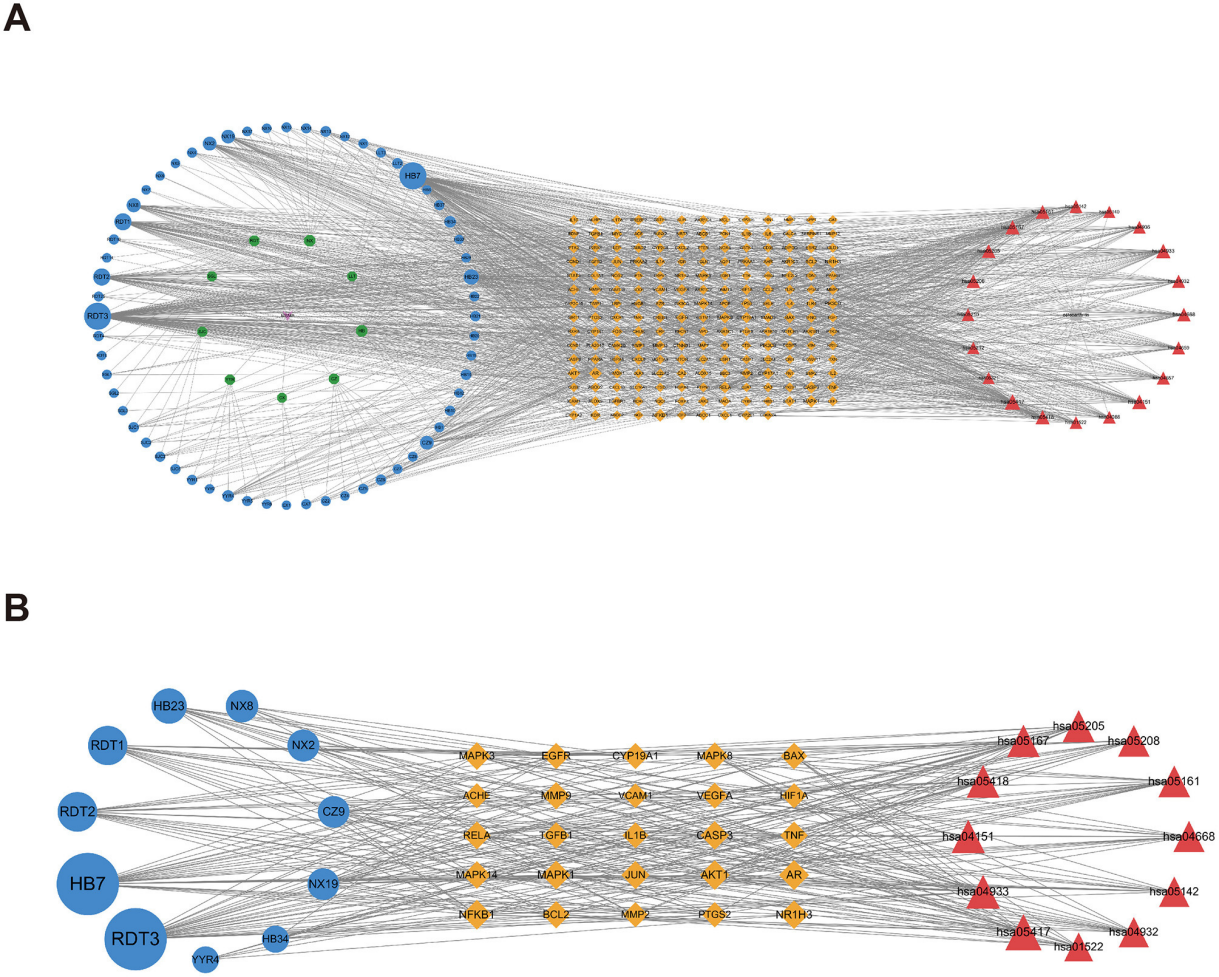
Compound–Component–Target–Pathway–Disease Network. Purple fusiform represents Modified Simiao Powder (MSMP), green hexagon represents Traditional Chinese medicine (TCM), blue circle represents medicinal chemical composition, yellow diamond represents intersection targets, and red triangle represents pathways. The figure size indicates the Degree value. **(A)** 283 nodes; **(B)** core nodes.

To elucidate the interrelationships among the various components of MSMP therapy for OA, we constructed a compound–component–target–pathway–disease network comprising 283 nodes, including one compound prescription name, nine Chinese herbal medicine names, 60 active components, 192 targets, 20 pathways, one disease name, and 1,454 edges (Figure 7A). We employed the “CentiScaPe 2.2” plugin to identify key active components, selecting those with a degree value at least twice the median and with CC and BC metrics near the median. Subsequently, we identified 11 central active components, 25 gene targets, and 12 pathways critical to the therapeutic process of MSMP for OA (Figure 7B). We selected the five highest-ranked bioactive constituents from TCM, along with their corresponding intersecting targets, for molecular docking studies (Table 2).

**Table 2:** Top 5 compounds and targets with medium values in the network diagram.

**Label**	**Name**	**Tape**	**Closeness unDir**	**Closeness unDir**	**Degree unDir**
HB7	Quercetin	mol	0.001968504	19,363.45552	133
RDT2	Luteolin	mol	0.001461988	7,401.726331	59
RDT1	Kaempferol	mol	0.001457726	3,321.147725	58
HB23	Berberine	mol	0.001440922	6,789.709352	45
CZ9	Wogonin	mol	0.001302083	1,267.345038	33
AR	Androgen receptor	gene	0.001490313	5,787.1636	28
AKT1	RAC-alpha serine/threonine-protein kinase	gene	0.001615509	1,813.687579	27
NFKB1	Nuclear factor NF-kappa-B p105 subunit	gene	0.001647446	2,183.444258	27
MAPK1	Mitogen-activated protein kinase 1	gene	0.001569859	1,274.584598	24
CASP3	Caspase-3	gene	0.001564945	1,402.231957	23

### 3.7 Molecular docking

**Figure 8:**
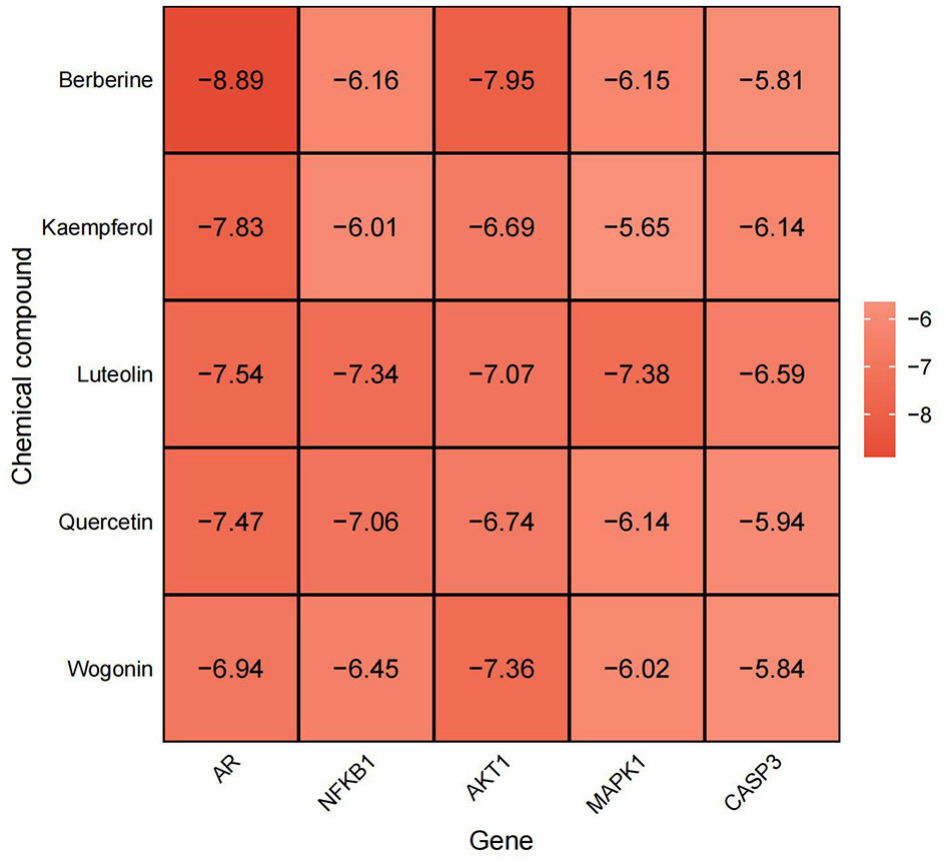
Heat map of molecular docking binding energy situation, abscissa represents Gene, ordinate represents chemical compound, darker color, lower value, represents more greater stability.

**Figure 9:**
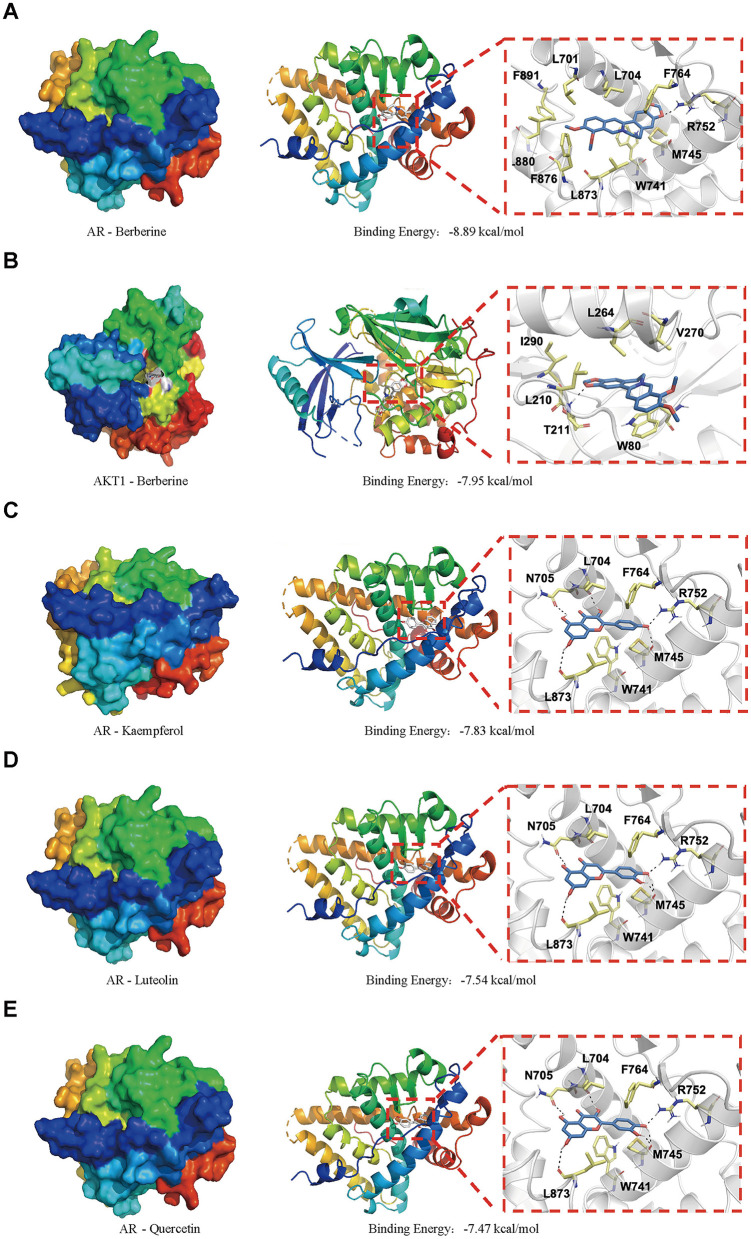
Molecular docking simulation results of the best target–ligand interactions in 2D and 3D images: **(A)** AR/berberine; **(B)** AKT1/berberine; **(C)** AR/kaempferol; **(D)** AR/luteolin; **(E)** AR/quercetin; The surface-active pocket structure is shown on the far left, the docking case in the middle, and the hydrogen bonding case is shown on the far right.

Molecular docking was performed to validate the principal active components of MSMP, namely quercetin, luteolin, kaempferol, berberine, and wogonin, against key potential therapeutic target proteins, such as the androgen receptor (AR), NFKB1, AKT1, MAPK1, and CASP3. Hydrogen bond formation was used as the criterion and the binding energies were computed. Heatmap visualizations of the results were generated using the ggplot2 package in R (Figure 8). The five groups with the lowest binding energies suggested that the main active compounds of MSMP established stable molecular interactions, such as hydrogen bonds, with the target receptor proteins (Figure 9). Berberine may interact with AR and AKT1. The AR-berberine docking model indicated that berberine primarily binds within the pocket through hydrophobic interactions and potentially forms hydrogen bonds with R752 (Figure 9A). In the AKT1/berberine docking model, hydrophobic amino acids, such as W80, L210, L264, and V270 of AKT1, stabilized berberine, whereas T211 formed hydrogen bonds with berberine (Figure 9B). These results suggest that kaempferol, luteolin, and quercetin may bind to AR. The docking results showed that the conformations of these three ligands within the complex were largely consistent, with the AR residues N705, R752, and L873 possibly forming hydrogen bonds to stabilize the ligand conformations (Figures 9C, D). Furthermore, the top five interaction models for each system exhibited consistent conformations, underscoring the reliability of the lowest energy models identified by molecular docking (Supplementary Figures S1, S2).

### 3.8 MD simulations

**Figure 10:**
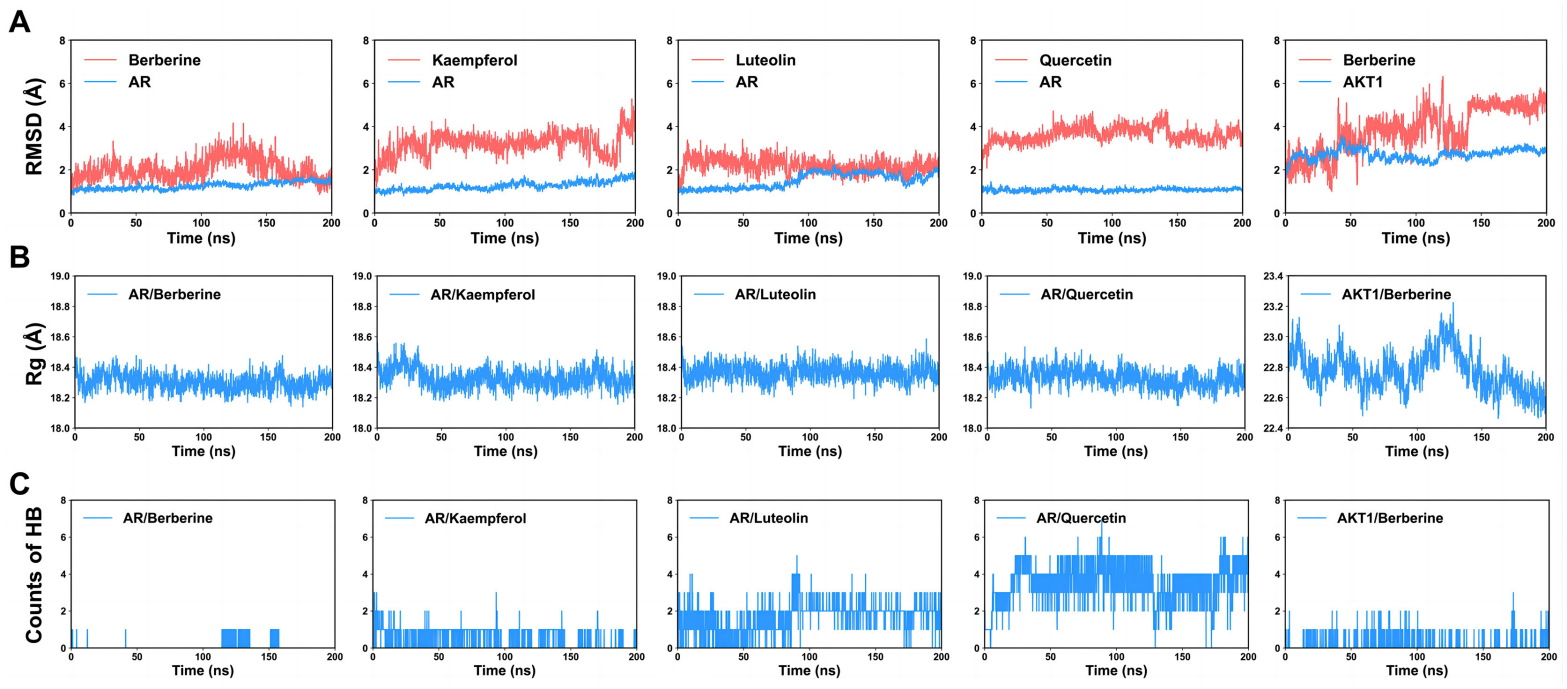
Molecular dynamics (MD) simulations of each protein–ligand complex. **(A)** Root-mean-square deviations (RMSDs) for each system (red lines: ligand, blue lines: protein). **(B)** Radius of gyration (Rg) of each protein–ligand complex. **(C)** Number of hydrogen bonds (HBs) between the ligand and the protein.

To assess the reliability and stability of the complex model predicted by molecular docking, we conducted 200-ns MD simulations for each model. Throughout the simulations, the ligands remained bound. For AR, all complexes demonstrated notable stability, as reflected by the stable root-mean-square deviation (RMSD) curves (Figure 10A). The RMSDs of the Cα atoms of AR were at or below 2 Å, suggesting that ligand binding did not significantly change the protein conformation. Radius of gyration (Rg) calculations also indicated the substantial stability of the androgen–ligand complexes (Figure 10B). Quercetin, with its five hydroxyl groups, formed the largest number of hydrogen bonds in the simulations (Figure 10C). The AKT1/berberine complex maintained its stability, particularly in the final 50-ns of the simulation, with a slight decrease in Rg, indicating a compact conformation (Figure 10B). These findings provide additional confirmation that all target–ligand complex models are stable and dependable.

**Figure 11:**
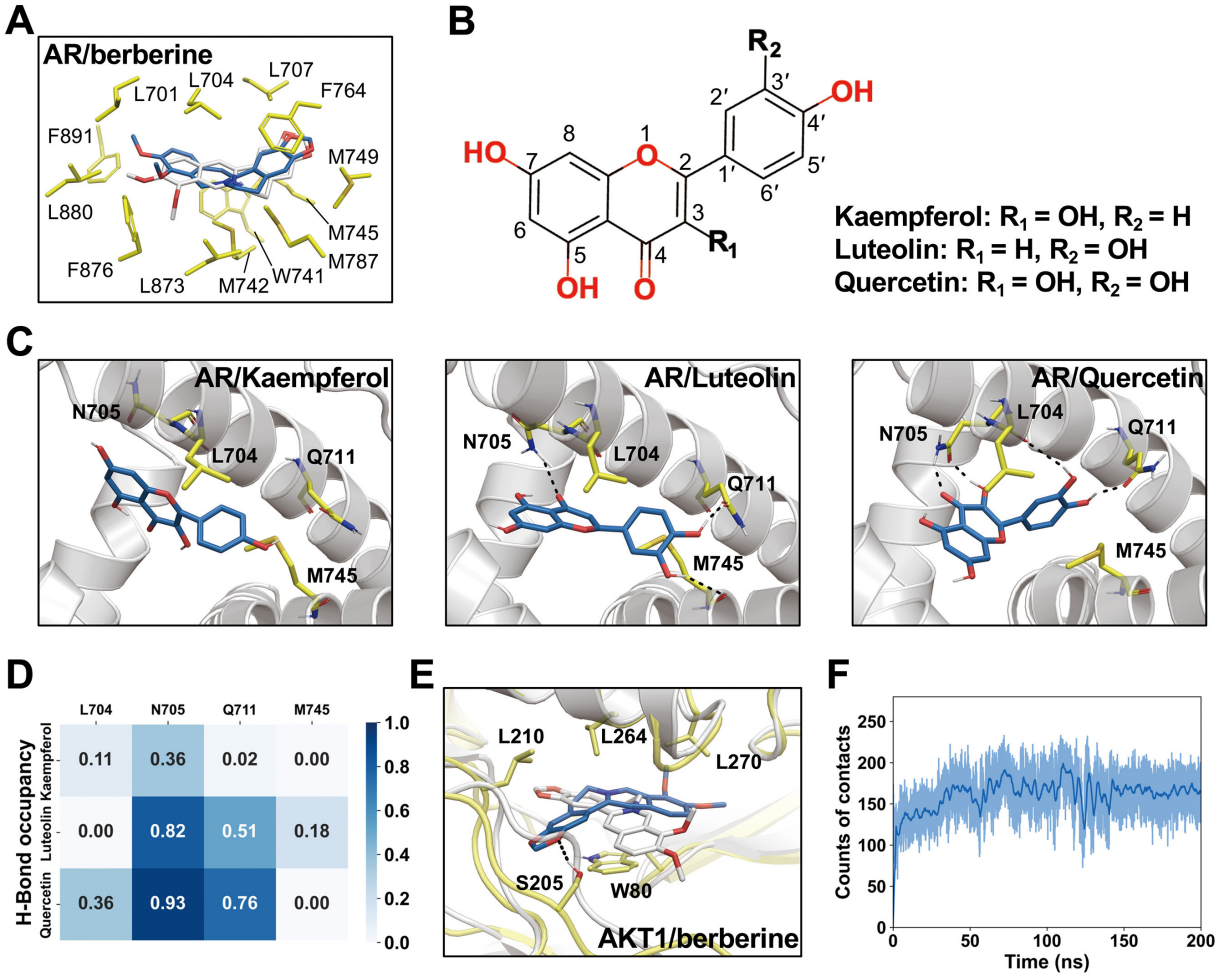
Interactions between ligands and receptors and representative conformations revealed by molecular dynamics (MD) simulations. **(A)** Representative conformation of AR/berberine complex from MD simulations. Berberine conformation obtained from molecular docking shown in white sticks. **(B)** Chemical structures of kaempferol, luteolin, and quercetin. **(C)** Representative conformations of AR/kaempferol, AR/luteolin, and AR/quercetin. **(D)** Occupancy of hydrogen bonds in MD simulations. **(E)** Representative conformation of AKR1/berberine obtained from MD simulations. **(F)** The counts of contacts between heavy atoms of berberine and AKT1 in MD simulations. A 4.5 Å distance cutoff was used for the calculations.

We further investigated the interactions between ligands and their targets using MD simulations. For AR/berberine, the ligand remained highly stable with minimal conformational changes throughout the MD simulation (RMSD_heavy − atom_ = 0.83 Å, Figure 11A). Kkaempferol, luteolin, and quercetin share the same core structure but differ in their substituents at the R1 and R2 positions, which may affect their interactions with AR (Figures 11B, C). Quercetin, with hydroxyl groups at both the R1 and R2 positions, can form hydrogen bonds with L704, N705, and Q711, resulting in higher hydrogen bond occupancy (Figure 11D), potentially indicating a higher affinity. Additionally, we observed noticeable conformational changes in the ligands during MD simulation, likely resulting from the smaller size and higher flexibility of kaempferol, luteolin, and quercetin than those of berberine. For AKT1/berberine, significant conformational changes were observed in the ligand; however, berberine remained bound within the pocket and formed new hydrogen bonds with S205 (Figure 11E). Contact number calculations indicated that the conformational changes in berberine established more interactions with AKT1 (Figure 11F), suggesting that the MD simulations captured a favorable binding mode.

## 4 Discussion

Currently, the integration of TCM with modern medical research emphasizes the clinical significance of integrative medicine, including natural products, in enhancing overall health outcomes. Integrative medicine, which combines traditional treatments with complementary therapies, is increasingly recognized for its potential to provide patient-centered, holistic care. Currently, numerous studies underscore the efficacy of integrative medical practices and their extensive clinical applications. Fattahi et al. (35) examined the Fine-Humor Producing Materia Medica in Persian Medicine and found that patients can benefit from fine-humor producing nutrition. A randomized double-blind placebo-controlled clinical trial by Meysami et al. (61) demonstrated that topical use of a cream comprising *Malva sylvestris* extract effectively reduced atopic dermatitis symptoms in children. Augustine et al. (36) reported that members of the Nutrition and Dietetics Association possess positive attitudes toward integrative medicine and are increasingly incorporating these practices into their professional routines. This shift indicates a rising interest and acceptance of integrative medicine among healthcare professionals, leading to its widespread adoption in contemporary medical practice. The increasing trend in integrative medicine and the increasing demand for natural products reflect this change. By embracing these therapies, healthcare providers can offer comprehensive and effective treatments, ultimately improving overall patient health outcomes.

In this study, we used network pharmacology to elucidate the potential molecular mechanisms of action of MSMP in the treatment of OA. Through the analysis of multiple databases and the filtration of intersecting target genes related to drugs used to treat OA, we identified nine natural drugs and their associated target genes. Employing Cytoscape software and a PPI network, we extracted 25 core targets from the compound–drug–ingredient–disease–target network. The core targets with degree values exceeding 20 were *AR, NFKB1, AKT1, MAPK1, CASP3, NR1H3, JUN*, and *RELA*. These high-ranking target genes are possibly crucial for the treatment of OA using MSMP. Simultaneously, these target proteins represent a notable research focus, and we aim to further analyze them in relation to OA. After adjusting for various risk factors, Fytili et al. (37) observed that women with LL genotypes for ER-beta and *AR* genes had a significantly increased risk of developing OA (*p* = 0.002 and 0.001). Therapeutically targeting NFKB1 emerges as a promising strategy to inhibit NF-κB signaling in the management of OA (38). Studies in animal models of OA have shown that bone spur formation is hindered in AKT1-deficient mice owing to the repression of nucleotide pyrophosphatase/phosphodiesterase 1 (39). MAPK1 overexpression mitigates IL-1β-induced inflammatory injury in chondrocytes (40). Caspase-3, a key protease involved in apoptosis (59), may play a major role in the inhibition of chondrocyte apoptosis (41). The modulation of LXR receptor activity (LXRα/NR1H3 and LXRβ/NR1H2) effectively alleviates joint discomfort in rat models with meniscal tears (42). JUN, which is regulated via the JNK–JUN signaling pathway, binds directly to the *Ncoa4* promoter and initiates transcription. This highlights the role of the JNK–JUN–NCOA4 axis in chondrocyte ferroptosis and the pathogenesis of OA and suggests that it is a potential therapeutic target (43). Nuclear RELA transcriptionally induces anti-apoptotic genes that are vital for chondrocyte survival. An increase in phosphorylated IKB-α levels and nuclear RELA levels correlate with cartilage deterioration, catabolism, and inflammatory molecule induction, accelerating the progression of OA (44). Therefore, these core targets are not only implicated in the pathogenesis of OA, but also represent potential therapeutic targets.

GO predictions were made for the treatment OA using MSMP in terms of BP, MF, and CC. The BP analysis indicated involvement in antioxidative responses, cellular reactions to chemical stress, and responses to reactive oxygen species. The CC analysis identified membrane rafts, membrane microdomains, and vesicles. The MF analysis implicated receptor ligand activity, signaling receptor activator activity, and DNA-binding transcription factor binding. These findings suggest that MSMP primarily attenuates the expression of inflammatory genes in OA via anti-inflammatory pathways. Interactions between proteins and active drug components modulate the oxidation of inflammatory factors, thereby influencing gene expression. This implied that the efficacy of MSMP in treating OA may be further substantiated by molecular docking and MD simulations.

Among the top 20 enriched pathways identified, the most significant were related to the AGE–RAGE signaling pathway implicated in diabetic complications. Other notable pathways included lipid metabolism and atherogenesis, fluid shear stress and its role in atherosclerosis, and TNF signaling. Additionally, pathways involved in Chagas disease, IL-17 signaling, infection by Kaposi's sarcoma-associated herpes virus, hepatitis B, and Th17 cell differentiation were also significantly enriched. Targeting the AGE–RAGE axis and its associated inflammatory and adhesive molecules may attenuate inflammation in the kidneys and systemically in murine models, as well as downregulate the TLR4/MyD88/NF-κB signaling pathway (45). In OA models, TLR4/MyD88/NF-κB axis disruption enhances cartilage integrity, diminishes apoptosis, and mitigates inflammation within synovial fluid (46). Dysregulated lipid metabolism and atherosclerosis may be the underlying contributors to OA (47). TNF-α and IL-1β have been extensively studied as key pro-inflammatory cytokines in the pathogenesis OA (48). Prevalent comorbidities among older individual patients with Chagas disease include osteoporosis, osteoarthritis, and dyslipidemia (49). In OA, IL-17 influences the inflammatory responses of chondrocytes and synovial fibroblasts, complement synthesis, hypoxic responses, angiogenesis, and glycolytic pathways (50). Therapeutic strategies targeting Th17 cells, IL-17 expression, IL-17 signaling pathway activation, and inflammatory mediator expression may be effective in the treatment of OA (51). In the context of viral arthritis, common viral etiologies include parvovirus, alphavirus, and hepatitis B (52), along with hepatitis C, Epstein–Barr virus, and tropical viruses, such as Zika and chikungunya viruses. However, Kaposi's sarcoma-associated herpes virus has not been linked to OA or MSMP, suggesting a potential avenue for future investigation. An imbalance in Th17 cells has been implicated in the progression of OA (53). Enrichment analysis revealed that multiple target genes, including *STAT3, STAT1, JUN, NFKB1, RELA*, and *MAPK1*, are involved in the regulation of Th17 cell differentiation, suggesting that MSMP may be effective at treating OA using a multi-gene, multi-pathway strategy.

Molecular docking studies were performed to substantiate the therapeutic efficacy of MSMP against OA. The top five protein–small molecule interactions were selected based on their degree values. Computer-simulated docking conformations revealed strong binding affinities between the active drug components and their target proteins. Berberine, kaempferol, luteolin, and quercetin demonstrated potent binding activities against AR and AKT1. Studies have confirmed that berberine mitigates OA-associated joint structural damage and pain in post-injury murine models by modulating AMPK (54). Kaempferol suppresses inflammation and extracellular matrix degradation in chondrocytes via the XIST/*miR-130a*/STAT3 pathway (55). Luteolin significantly reduces H_2_O_2_-induced cell death and apoptosis in primary mouse chondrocytes and notably inhibits OA progression in mouse models (56). Quercetin has emerged as a novel therapeutic candidate for OA, acting via diverse mechanisms, including the modulation of inflammatory cytokines, such as IL-1β, TNF-α, IL-6, and IL-10; the inhibition of oxidative stress; the attenuation of cartilage ECM degradation; and a decrease in chondroitin sulfate 4 levels (57).

MD simulations were employed to further investigate the behavior and stability of these binding complexes over time. Our observations indicated that all complexes exhibited significant stability, particularly luteolin and AR, which may provide insights for the development of novel therapeutics. Each complex was found to form hydrogen bonds, with quercetin forming the most. Previous studies have suggested that the formation of intramolecular hydrogen bonds can positively influence the triad of drug permeability, solubility, and potency (58). Through MD simulations, we can observe dynamic changes, conformational adaptations, and interaction forces between proteins and ligands, thereby validating the biological significance and practical feasibility of the docking results.

Extensive experiments have demonstrated that multiple active compounds identified using network pharmacology have potential as candidate drugs for the treatment of OA, offering promising directions for novel drug discovery. This screening process not only elucidates the active constituents of natural plants used in disease therapy, but also aids in the refinement of herbal extracts into simple and potent medicinal components. Therefore, we suggest that MSMP delivers therapeutic benefits in OA via bioactive molecules, related signaling pathways, and targeted genes.

## 5 Conclusion

We used network pharmacology, molecular docking, and MD simulations to elucidate the mechanism by which MSMP modulates OA using multi-molecular, multi-pathway, and multi-target strategies. Our findings suggest that compounds in MSMP, including berberine, kaempferol, luteolin, quercetin, and wogonin, can optimally bind to pivotal proteins, such as AR, NFKB1, AKT1, MAPK1, and CASP3. This reinforces the scientific rationale for the therapeutic effects of MSMP against OA, provides a crucial reference for novel drug development, and lays the theoretical groundwork for exploring key genes and mechanisms of drug action in the treatment of OA. In addition, this study offers remarkable insights into the clinical use of MSMP.

## Data Availability

The original contributions presented in the study are included in the article/Supplementary material, further inquiries can be directed to the corresponding author.

## References

[1] Childs BG Gluscevic M Baker DJ Laberge RM Marquess D Dananberg J. Senescent cells: an emerging target for diseases of ageing. Nat Rev Drug Discov. (2017) 16:718–35. 10.1038/nrd.2017.116 28729727 PMC5942225

[2] Abramoff B Caldera FE. Osteoarthritis: pathology, diagnosis, and treatment options. Med Clin North Am. (2020) 104:293–311. 10.1016/j.mcna.2019.10.007 32035570

[3] Boer CG Radjabzadeh D Medina-Gomez C Garmaeva S Schiphof D Arp P. Intestinal microbiome composition and its relation to joint pain and inflammation. Nat Commun. (2019) 10:4881. 10.1038/s41467-019-12873-4 31653850 PMC6814863

[4] Ling C Zhang Y Li J Chen W Ling C. Clinical use of toxic proteins and peptides from tian hua fen and scorpion venom. Curr Protein Pept Sci. (2019) 20:285–95. 10.2174/1389203719666180622100641 29932034

[5] Dashtdar M Dashtdar MR Dashtdar B Kardi K Shirazi MK. The concept of wind in traditional Chinese medicine. J Pharmacopuncture. (2016) 19:293–302. 10.3831/KPI.2016.19.030 28097039 PMC5234349

[6] Wang C Gao Y Zhang Z Chi Q Liu Y Yang L. Safflower yellow alleviates osteoarthritis and prevents inflammation by inhibiting PGE2 release and regulating NF-κb/sirt1/AMPK signaling pathways. Phytomedicine. (2020) 78:153305. 10.1016/j.phymed.2020.153305 32871523

[7] Xiang C Liao Y Chen Z Xiao B Zhao Z Li A. Network pharmacology and molecular docking to elucidate the potential mechanism of ligusticum chuanxiong against osteoarthritis. Front Pharmacol. (2022) 13:854215. 10.3389/fphar.2022.854215 35496280 PMC9050356

[8] Luo E Zhang D Luo H Liu B Zhao K Zhao Y. Treatment efficacy analysis of traditional Chinese medicine for novel coronavirus pneumonia (COVID-19): an empirical study from Wuhan, Hubei Province, China. Chin Med. (2020) 15:34. 10.1186/s13020-020-00317-x 32308732 PMC7156896

[9] Zhang P Zhang D Zhou W Wang L Wang B Zhang T. Network pharmacology: towards the artificial intelligence-based precision traditional Chinese medicine. Brief Bioinform. (2023) 25:bbad518. 10.1093/bib/bbad518 38197310 PMC10777171

[10] Li S. Network pharmacology evaluation method guidance-draft. World J Tradit Chin Med. (2021) 7:146. 10.4103/wjtcm.wjtcm_11_21

[11] Li S. Possible Correlation between TCM Syndromes and Molecular Network Regulation Mechanism. Hangzhou: The First Annual Conference of China Association for Science and Technology (1999).

[12] Li S Wang YY Ji L. A discussion and case study of complexities in traditional Chinese medicine. J Syst Simul. (2002) 14:1429–1403.

[13] Hopkins AL. Network pharmacology. Nat Biotechnol. (2007) 25:1110–1. 10.1038/nbt1007-1110 17921993

[14] Niu M Zhang SQ Zhang B Yang K. Interpretation of network pharmacology evaluation method Guidance. Chin Tradit Herbal Drugs. (2021) 52:4119–29. 10.7501/j.issn.0253-2670.2021.14.001

[15] Pinzi L Rastelli G. Molecular docking: shifting paradigms in drug discovery. Int J Mol Sci. (2019) 20:4331. 10.3390/ijms20184331 31487867 PMC6769923

[16] Ji P Zhao NS Wu FL Wei YM Laba CD Wujin CM. Mechanisms predictive of Tibetan Medicine Sophora moorcroftiana alkaloids for treatment of lung cancer based on the network pharmacology and molecular docking. BMC Complement Med Ther. (2024) 24:47. 10.1186/s12906-024-04342-3 38245694 PMC10799429

[17] Rehman AU Zhen G Zhong B Ni D Li J Nasir A. Mechanism of zinc ejection by disulfiram in nonstructural protein 5A. Phys Chem Chem Phys. (2021) 23:12204–15. 10.1039/D0CP06360F 34008604

[18] Nasir A Samad A Ullah S Ali A Wei DQ Qian B. Omicron variant (B.1.1.529) challenge the integrity of blood brain barrier: Evidence from protein structural analysis. Comput Biol Med. (2024) 169:107906. 10.1016/j.compbiomed.2023.107906 38154156

[19] Ciancetta A Sabbadin D Federico S Spalluto G Moro S. Advances in Computational Techniques to Study GPCR-Ligand Recognition. Trends Pharmacol Sci. (2015) 36:878–90. 10.1016/j.tips.2015.08.006 26538318

[20] Fan JH Xu MM Zhou LM Gui ZW Huang L Li XG. Integrating network pharmacology deciphers the action mechanism of Zuojin capsule in suppressing colorectal cancer. Phytomedicine. (2022) 96:153881. 10.1016/j.phymed.2021.153881 34942456

[21] Daina A Michielin O Zoete V. SwissADME: a free web tool to evaluate pharmacokinetics, drug-likeness and medicinal chemistry friendliness of small molecules. Sci Rep. (2017) 7:42717. 10.1038/srep42717 28256516 PMC5335600

[22] Yu G Wang LG Han Y He QY. clusterProfiler: an R package for comparing biological themes among gene clusters. Omics J Integr Biol. (2012) 16:284–7. 10.1089/omi.2011.0118 22455463 PMC3339379

[23] Berman HM Westbrook J Feng Z Gilliland G Bhat TN Weissig H. The protein data bank. Nucleic Acids Res. (2000) 28:235–42. 10.1093/nar/28.1.235 10592235 PMC102472

[24] Seeliger D de Groot BL. Ligand docking and binding site analysis with PyMOL and Autodock/Vina. J Comput Aided Mol Des. (2010) 24:417–422. 10.1007/s10822-010-9352-6 20401516 PMC2881210

[25] O'Boyle NM Banck M James CA Morley C Vandermeersch T Hutchison GR. Open Babel: an open chemical toolbox. J Cheminform. (2011) 3:33. 10.1186/1758-2946-3-33 21982300 PMC3198950

[26] He Q Liu C Wang X Rong K Zhu M Duan L. the mechanism of curcumin in the treatment of colon cancer based on network pharmacology and molecular docking. Front Pharmacol. (2023) 14:1102581. 10.3389/fphar.2023.1102581 36874006 PMC9975159

[27] Maier JA Martinez C Kasavajhala K Wickstrom L Hauser KE Simmerling C. ff14SB: Improving the accuracy of protein side chain and backbone parameters from ff99SB. J Chem Theory Comput. (2015) 11:3696–713. 10.1021/acs.jctc.5b00255 26574453 PMC4821407

[28] Wang J Wolf RM Caldwell JW Kollman PA Case DA. Development and testing of a general amber force field. J Comput Chem. (2004) 25:1157–74. 10.1002/jcc.20035 15116359

[29] Schauperl M Nerenberg PS Jang H Wang LP Bayly CI Mobley DL. Non-bonded force field model with advanced restrained electrostatic potential charges (RESP2). Commun Chem. (2020) 3:44. 10.1038/s42004-020-0291-4 34136662 PMC8204736

[30] Lu T Chen F. Multiwfn: a multifunctional wavefunction analyzer. J Comput Chem. (2012) 33:580–92. 10.1002/jcc.22885 22162017

[31] Van Der Spoel D Lindahl E Hess B Groenhof G Mark AE Berendsen HJ. GROMACS: fast, flexible, and free. J Comput Chem. (2005) 26:1701–18. 10.1002/jcc.20291 16211538

[32] Hess B Bekker H Berendsen HJC Fraaije J. LINCS: a linear constraint solver for molecular simulations. J Comput Chem. (1997) 18:1463–72. 10.1002/(SICI)1096-987X(199709)18:12<1463::AID-JCC4>3.3.CO;2-L

[33] Bussi G Donadio D Parrinello M. Canonical sampling through velocity rescaling. J Chem Phys. (2007) 126:014101. 10.1063/1.2408420 17212484

[34] Bernetti M Bussi G. Pressure control using stochastic cell rescaling. J Chem Phys. (2020) 153:114107. 10.1063/5.0020514 32962386

[35] Fattahi YZ Fadaei F Asghari A Naghizadeh A Karimi M. Fine-humor producing materia medica in persian medicine. Tradit Integr Med. (2022) 7:244–53. 10.18502/tim.v7i2.992724719702

[36] Augustine MB Swift KM Harris SR Anderson EJ. Integrative medicine: education, perceived knowledge, attitudes, and practice among academy of nutrition and dietetics members. J Acad Nutr Diet. (2016) 116:319–29. 10.1016/j.jand.2015.08.015 26387076

[37] Fytili P Giannatou E Papanikolaou V Stripeli F Karachalios T Malizos K. Association of repeat polymorphisms in the estrogen receptors alpha, beta, and androgen receptor genes with knee osteoarthritis. Clin Genet. (2005) 68:268–77. 10.1111/j.1399-0004.2005.00495.x 16098017

[38] Tang S Nie X Ruan J Cao Y Kang J Ding C. Circular RNA circNFKB1 promotes osteoarthritis progression through interacting with ENO1 and sustaining NF-κB signaling. Cell Death Dis. (2022) 13:695. 10.1038/s41419-022-05148-2 35945200 PMC9363463

[39] Fukai A Kawamura N Saito T Oshima Y Ikeda T Kugimiya F. Akt1 in murine chondrocytes controls cartilage calcification during endochondral ossification under physiologic and pathologic conditions. Arthritis Rheum. (2010) 62:826–36. 10.1002/art.27296 20187155

[40] Hu X Ji X Yang M Fan S Wang J Lu M. Cdc42 is essential for both articular cartilage degeneration and subchondral bone deterioration in experimental osteoarthritis. J Bone Miner Res. (2018) 33:945–58. 10.1002/jbmr.3380 29314205

[41] Wang BW Jiang Y Yao ZL Chen PS Yu B Wang SN. Aucubin protects chondrocytes against IL-1β-induced apoptosis in vitro and inhibits osteoarthritis in mice model. Drug Des Devel Ther. (2019) 13:3529–38. 10.2147/DDDT.S210220 31631977 PMC6791845

[42] Li N Rivéra-Bermúdez MA Zhang M Tejada J Glasson SS Collins-Racie LA. modulation blocks prostaglandin E2 production and matrix degradation in cartilage and alleviates pain in a rat osteoarthritis model. Proc Natl Acad Sci U S A. (2010) 107:3734–9. 10.1073/pnas.0911377107 20133709 PMC2840473

[43] Sun K Hou L Guo Z Wang G Guo J Xu J. JNK-JUN-NCOA4 axis contributes to chondrocyte ferroptosis and aggravates osteoarthritis via ferritinophagy. Free Radic Biol Med. (2023) 200:87–101. 10.1016/j.freeradbiomed.2023.03.008 36907253

[44] Kobayashi H Chang SH Mori D Itoh S Hirata M Hosaka Y. Biphasic regulation of chondrocytes by Rela through induction of anti-apoptotic and catabolic target genes. Nat Commun. (2016) 7:13336. 10.1038/ncomms13336 27830706 PMC5109547

[45] Chen J Peng H Chen C Wang Y Sang T Cai Z. NAG-1/GDF15 inhibits diabetic nephropathy via inhibiting AGE/RAGE-mediated inflammation signaling pathways in C57BL/6 mice and HK-2 cells. Life Sci. (2022) 311:121142. 10.1016/j.lfs.2022.121142 36367498

[46] Cai D Zhang J Yang J Lv Q Zhong C. Overexpression of FTO alleviates osteoarthritis by regulating the processing of miR-515-5p and the TLR4/MyD88/NF-κB axis. Int Immunopharmacol. (2023) 114:109524. 10.1016/j.intimp.2022.109524 36538851

[47] Gkretsi V Simopoulou T Tsezou A. Lipid metabolism and osteoarthritis: lessons from atherosclerosis. Prog Lipid Res. (2011) 50:133–40. 10.1016/j.plipres.2010.11.001 21115041

[48] Yu H Huang T Lu WW Tong L Chen D. Osteoarthritis pain. Int J Mol Sci. (2022) 23:4642. 10.3390/ijms23094642 35563035 PMC9105801

[49] Alves RM Thomaz RP Almeida EA Wanderley Jda S Guariento ME. Chagas' disease and ageing: the coexistence of other chronic diseases with Chagas' disease in elderly patients. Rev Soc Bras Med Trop. (2009) 42:622–8. 10.1590/S0037-86822009000600002 20209343

[50] Mimpen JY Baldwin MJ Cribbs AP Philpott M Carr AJ Dakin SG. Interleukin-17A causes osteoarthritis-like transcriptional changes in human osteoarthritis-derived chondrocytes and synovial fibroblasts in vitro. Front Immunol. (2021) 12:676173. 10.3389/fimmu.2021.676173 34054865 PMC8153485

[51] Xiao J Zhang P Cai FL Luo CG Pu T Pan XL. IL-17 in osteoarthritis: a narrative review. Open Life Sci. (2023) 18:20220747. 10.1515/biol-2022-0747 37854319 PMC10579884

[52] Tiwari V Bergman MJ. Viral Arthritis. 2023 Jul 4. In: *StatPearls [Internet]*. Treasure Island (FL): StatPearls Publishing (2024). 30285402

[53] Ye X Lu Q Yang A Rao J Xie W He C. MiR-206 regulates the Th17/Treg ratio during osteoarthritis. Mol Med. (2021) 27:64. 10.1186/s10020-021-00315-1 34147072 PMC8214293

[54] Li J Wang Y Chen D Liu-Bryan R. Oral administration of berberine limits post-traumatic osteoarthritis development and associated pain via AMP-activated protein kinase (AMPK) in mice. Osteoarthr Cartilage. (2022) 30:160–71. 10.1016/j.joca.2021.10.004 34687898 PMC8712393

[55] Xiao Y Liu L Zheng Y Liu W Xu Y. Kaempferol attenuates the effects of XIST/miR-130a/STAT3 on inflammation and extracellular matrix degradation in osteoarthritis. Future Med Chem. (2021) 13:1451–64. 10.4155/fmc-2021-0127 34120462

[56] Zhou Z Zhang L Liu Y Huang C Xia W Zhou H. Luteolin protects chondrocytes from H2O2-induced oxidative injury and attenuates osteoarthritis progression by activating AMPK-Nrf2 signaling. Oxid Med Cell Longev. (2022) 2022:5635797. 10.1155/2022/5635797 35154568 PMC8825676

[57] Samadi F Kahrizi MS Heydari F Arefnezhad R Roghani-Shahraki H Mokhtari Ardekani A. Quercetin and osteoarthritis: a mechanistic review on the present documents. Pharmacology. (2022) 107:464–71. 10.1159/000525494 35793647

[58] Caron G Kihlberg J Ermondi G. Intramolecular hydrogen bonding: An opportunity for improved design in medicinal chemistry. Med Res Rev. (2019) 39:1707–29. 10.1002/med.21562 30659634

[59] Aizawa T Kon T Einhorn TA Gerstenfeld LC. Induction of apoptosis in chondrocytes by tumor necrosis factor-alpha. J Orthop Res. (2001) 19:785–96. 10.1016/S0736-0266(00)00078-4 11562122

[60] Dror RO Dirks RM Grossman JP Xu H. Biomolecular simulation: a computational microscope for molecular biology. Annu Rev Biophys. (2012) 41:429–52. 10.1146/annurev-biophys-042910-155245 22577825

[61] Meysami M Hashempur MH Kamalinejad M Emtiazy M. Efficacy of short term topical Malva sylvestris L. cream in pediatric patients with atopic dermatitis: a randomized double-blind placebo-controlled clinical trial. Endocr Metab Immune Disord Drug Targets. (2021) 21:1673–8. 10.2174/1871530320666201023125411 33100212

[62] Reliability and reproducibility checklist for molecular dynamics simulations. Commun Biol. (2023) 6:268. 10.1038/s42003-023-04653-0 36918708 PMC10014944

