# Supplementary material for: Exploring the mechanism of action of Modified Simiao Powder in the treatment of osteoarthritis: an in-silico study
Source: Front Med (Lausanne). 2024 Oct 18;11:1422306. doi: 10.3389/fmed.2024.1422306 (PMC11527633; doi:10.3389/fmed.2024.1422306)
Supplement: Supplementary file 9 [file Table_9.DOCX]

Supplementary Material

# Supplementary Data

The Supplementary Data for this article can be found online at:

# Supplementary Figures and Tables

## Supplementary Figures

**Supplementary Figure 1.** Top five complex models predicted by AutoDock

**Supplementary Figure 2.** Heatmap of binding energies for the top five complex models predicted by AutoDock, with numerical annotations.
