# Supplementary figures and images for: Exploring the mechanism of action of Modified Simiao Powder in the treatment of osteoarthritis: an in-silico study
Source: Front Med (Lausanne). 2024 Oct 18;11:1422306. doi: 10.3389/fmed.2024.1422306 (PMC11527633; doi:10.3389/fmed.2024.1422306)

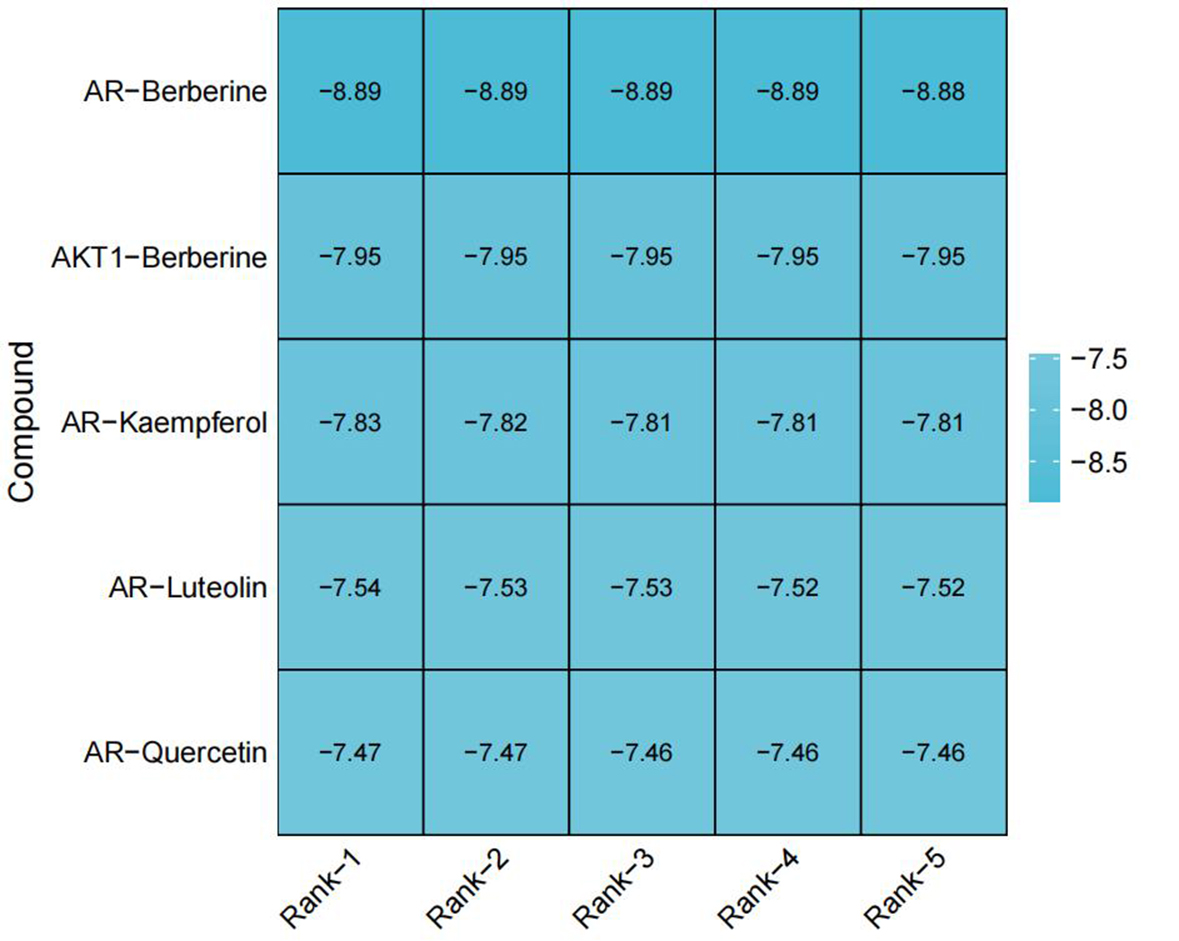

Supplement: Supplementary file 12 [file Image_2.JPEG]
